# A randomized control trial comparing the crestal bone loss between short, wide diameter implants, long and narrow diameter implants with different crown: Implant ratio - A preliminary report

**DOI:** 10.6026/973206300213206

**Published:** 2025-09-30

**Authors:** Ilangkumaran R., Srinivasan J., Manoharan P.S., Fahd N. AL Qahtani, Karthi Arivarasan N., Manoj M.

**Affiliations:** 1 Department of Prosthodontics, Mahatma Gandhi Postgraduate Institute of Dental Sciences, Puducherry, Tamilnadu, India; 2 Department of Prosthodontics, Faculty of Dentistry, Al-Baha University, Alaqiq, Saudi Arabia; 3 Department of Prosthodontics, Saveetha University, Chennai, Tamil Nadu, India; 4 Department of Dentistry, Al-Baha University, Alaqiq, Saudi Arabia; 5 Department of Prosthodontics, Mahatma Gandhi Postgraduate Institute of Dental Sciences, Puducherry, Tamil Nadu, India; 6 Department of Periodontics, Mahatma Gandhi Postgraduate Institute of Dental Sciences, Puducherry, Tamil Nadu, India

**Keywords:** Randomized control trial, short wide implants, crestal bone loss, crown: implant ratio, two-stage implant placement

## Abstract

The effect of implant size and crown-to-implant (C/I) ratio on crestal bone loss in partially edentulous patients is of interest. Fourteen patients were assigned to short, wide implant with high C/I ratio (1-1.2) or long, narrow implants with low C/I ratio (0.5-0.9). Crestal bone loss was assessed radiographically at baseline and four months post-loading using standardized imaging and blinded observers. Mean bone loss was 0.232 mm for high C/I implants and 0.459 mm for low C/I implants, with no significant difference (p = 0.133). These findings suggest that higher C/I ratios do not adversely affect short-term implant performance in the absence of mechanical overload.

## Background:

Dental implants are now a popular treatment modality for replacing missing teeth, with high survival rates and predictable long-term results relative to traditional fixed prostheses. Long-term implant success is not just based on osseointegration but also the maintenance of peri-implant crestal bone, which is an important marker of implant health [1]. Prior criteria that have been espoused by the International Congress of Oral Implantologists highlight that crestal bone stability, together with pain, mobility, and lack of infection, are the focus of the definition of success of the implant [2]. Clinical examination indicates that implants can experience marginal bone remodeling within the first year of function, followed by an annual loss of bone of around 0.2 mm subsequently [3]. Crestal bone loss is controlled by various factors, grouped broadly into implant-related and patient-related variables. Patient-related variables are systemic conditions, bone density, and oral health, while implant-related variables involve implant design, diameter, length, surface quality, and crown-to-implant (C/I) ratio [4]. Of these, the C/I ratio, defined as the ratio between crown height and implant length, has been controversial. A high C/I ratio is usually biomechanically unfavorable because of large leverage forces, non-axial loading, and possible overload of the bone implant interface [5]. Finite element analyses and photoelastic stress studies demonstrate that a larger implant diameter decreases stress concentrations at the crestal bone, which may counteract the adverse effects of the reduced length with wide-diameter implants [6].

The clinical significance of C/I ratio is still uncertain. Retrospective studies have also suggested threshold values, with some indicating that ratios greater than 1:1 might compromise results, and others showing no correlation with implant survival or crestal bone stability [7]. In addition, these studies are often statistically significant as a result of large sample sizes, but contain relatively small numbers of implants having high C/I ratios, making them not generalizable. Later systematic reviews and meta-analyses have stressed that crown height space (CHS) is perhaps a more important factor than C/I ratio itself in determining biomechanical load distribution. Significantly, these reviews also point out that the majority of evidence available is based on retrospective or observational studies with relatively few well-designed randomized controlled trials (RCTs). The availability of short implants with highly developed surface treatments has presented a plausible option compared to bone grafting in cases of compromised ridge height. Although shorter implants automatically mean greater C/I ratios, their ability to match the performance of longer implants is yet to be fully explored in possible clinical situations [8]. It is important that this deficiency be addressed to help guide clinicians through treatment planning for patients with short vertical bone. Therefore, it is of interest to investigate whether short wide-diameter implants with higher C/I ratios perform comparably to long narrow-diameter implants with lower ratios in terms of crestal bone loss, under conditions of constant crown height space.

## Materials and Methods:

This is a prospective, parallel-group randomized controlled trial that was conducted at Indira Gandhi Institute of Dental Sciences, Puducherry, India. Approval from the Institutional Review Board and Ethics Committee was obtained, and all procedures adhered to the Declaration of Helsinki. Fourteen partially edentulous patients were recruited to the study between the ages of 23 and 50 years with healed mandibular ridges and a crown height space of 10-11 mm, after all patients provided written informed consent. Any patients with systemic disease, poor oral hygiene, parafunctional habits, or poor ridge anatomy were excluded to reduce confounding variables. Eligible patients were randomly assigned to two groups using computer-generated randomization and allocation concealment. Group 1 received short wide diameter implants with a high crown-to-implant (C/I) ratio (1-1.2) and Group 2 received long narrow diameter implants with a low C/I ratio (0.5-0.9). All implants were placed by a single experienced operator using standardized two-stage surgical protocols while under antibiotic prophylaxis; their initial surgical positions were checked using acrylic stents to maintain the same position at surgery for all patients. Once a healing period of three months had elapsed, all implants were restored with single-unit cement-retained crowns. On the day of prosthetic loading, radiovisiography (RVG) images were taken to establish baseline levels and repeated after 4 months in a standardized manner with the same exposure parameters and beam positioning devices. On all radiographs, the area to be quantified was calibrated by implant dimensions. All images were analyzed in Adobe Photoshop templates and UTHSCSA Image Tool software (University of Texas Health Science Center at San Antonio, TX); all crestal bone level assessments were performed independently by two blinded and calibrated examiners along the mesial and distal surfaces of each implant. Disagreements were resolved by consensus. Crestal bone loss was defined as the change between baseline measurement and follow-up measurement and recorded with an accuracy of 0.01 mm. Data were compiled in Microsoft Excel and analyzed in SPSS version 19.0 (IBM, US). Normality assessment was performed and since the data were found to be non-parametric; the Mann-Whitney U test was used to compare mean crestal bone loss between groups. The sample size calculation had established that 10 subjects per group would achieve sufficient power, therefore, there were 2 extraneous participants added to each group to counterbalance potential dropouts. A level of statistical significance was established at p < 0.05.

## Results:

Fourteen partially edentulous patients volunteered for the study. The volunteers were evenly randomized to two groups of seven where two patients were lost to follow-up (one in each group), resulting in 11 patients completing follow-up in each group. All patients were restored with single-unit cement-retained crowns in the mandibular posterior region, and no patients experienced failures of the implants during the study. These preliminary findings suggest that both short wide and long narrow implants can be expected to perform comparably in terms of early crestal bone loss under similar crown height space restrictions, with a non-significant trend towards the shorter implants as the better performing implant. At four-months post-loading, the mean crestal bone loss for Group 1 (Short wide-diameter implants, with a high C/I ratio of 1 to 1.2) is 0.232 ± 0.234 mm; Group 2 (Long narrow-diameter implants, with low C/I ratio of 0.5 to 0.9) had a mean crestal bone loss of 0.459 ±0.296 mm. The mean difference between Group 1 and Group 2 was -0.227 mm; again, the short wide implants performed better than the long narrow implants. However, the difference was not statistically significant (p = 0.133). Table 1 (see PDF) shows the baseline demographic and clinical characteristics of the study participants in both groups. All patients were systemically healthy (ASA I or II), with comparable age ranges, ridge dimensions, and crown height space, indicating that the two groups were similar at baseline before intervention. Table 2 (see PDF) shows the comparative analysis of crestal bone loss between the two groups using the Mann-Whitney U test. Figure 1 (see PDF) shows enrollment, randomization, allocation, follow-up, and analysis of study participants. Table 1 (see PDF) shows the baseline demographic and clinical characteristics of the study participants. Both groups were comparable in terms of age range, ridge height, ridge width, crown height space, implant site, and systemic health status (ASA I or II), ensuring uniform baseline conditions prior to intervention. Table 2 (see PDF) shows the comparative analysis of crestal bone loss between the two groups using the Mann-Whitney U test. Group 1 (short wide-diameter implants with high C/I ratio) demonstrated a mean crestal bone loss of 0.232 mm, while Group 2 (long narrow-diameter implants with low C/I ratio) showed a mean loss of 0.459 mm; the difference of -0.227 mm was not statistically significant (p = 0.133).

## Discussion:

This randomized controlled trial measured the impact of implant diameter and crown-to-implant ratio (C/I) on crestal bone loss on the implant site in compromised partially edentulous patients, under idealized crown height space conditions, and noted a statistically insignificant amount of crestal bone loss for short broad diameter implants with greater C/I ratios versus long narrow diameter implants with less C/I ratios four months after loading [9]. Although bone loss was slightly less for the short broad diameter implant at four months, the differences were not statistically significant, indicating both implants performed similarly in the short loading period. The results reveal that an increased C/I ratio does not necessarily cause an adverse effect on short-term implant outcomes when the crown height space is held constant [10]. This presents further evidence that the nay singular aspect of an implant, such as length, is not the only variable influencing early crestal bone remodeling. Instead, implant diameter and stress distribution patterns are likely much more important variables influencing peri-implant bone stability than the length of the implant [11]. Wider diameter implants may help dissipate functional stresses at the implant-bone interface, which is particularly important at the crestal level, where resorption is most likely to occur [12]. This biomechanical rationale could explain the approach to limit bone loss with the short, wide implants in this study, particularly with the C/I ratios in the wide group being higher [13]. There also may be biological reasons for this finding. Preserving crestal bone is not determined just by mechanical stress, but the establishment of a biologic width, health of the peri-implant soft tissue, and inflammatory response also play an important role. This brings up the point that this study controlled for surgical protocol, type of implant, or crown height space as confounding variable and thus limited the biological influence on any of the implants. Based on the C/I ratio ranges (0.5-0.9 and 1.0-1.2), peri-implant tissue appeared to adapt successfully relative to the C/I, without any negative outcomes in the short-term. The impact of C/I ratios outside of this range is unknown, or the impact of C/I ratios outside of this range over a longer follow-up period is not yet known [14]. This study has significant clinical implications. For patients with limited vertical bone height, bone grafting procedures are often necessary to accommodate longer implants, which invariably increases costs, morbidity, and overall treatment time. The comparable outcomes of short wide-diameter implants used in this study suggest these implants can be a valid alternative since they are less invasive and offer similar clinical outcomes. This further supports the mounting evidence in the literature that short implants can lead to predictable outcomes in cases of decreased ridge height and in some cases can lead to avoidance of grafting procedures altogether [15].

The study design has strengths (such as being randomized, using standardized protocols, and producing blinded radiographic outcomes) but there are limitations to consider. The follow-up of four months is relatively short and makes it difficult to assess long-term crestal bone stability. The small sample size limits the statistical power of the study, and as such, clinical trials should be interpreted with caution as small sample sizes in a study can result in failure to detect subtle differences between groups. Additionally, two different ranges of C/I ratio were evaluated so there is uncertainty regarding outcomes when the C/I ratio is more extreme. Lastly, patient-related variables (gender distribution, occlusal loading patterns, and parafunctional habits) were not collected in this study, which may limit the generalizability of the study. Larger sample sizes, multi-centered studies, and longer follow-up times in future studies will allow for better evaluation of the long-term performance of implants across a wider spectrum of C/I ratios. Other prosthetic and biomechanical parameters, including implant angulation, abutment design, occlusal table width, and patient-specific functional habits, must be assessed to provide a clearer picture of all the factors impacting peri-implant crestal bone stability.

## Conclusion:

We show that short wide-diameter implants with higher crown-to-implant ratios have comparably high success in early crestal bone loss, with no statistically significant difference in early crestal bone loss, as compared to long narrow-diameter implants with lower crown-to-implant ratios. Considering these results, including that short implants may be a viable alternative to grafting procedures in patients with limited ridge height, further long-term studies and larger trials are warranted to substantiate long-term success equivalence.
